# Voltage-dependent plasticity of spin-polarized conductance in phenyl-based single-molecule magnetic tunnel junctions

**DOI:** 10.1371/journal.pone.0257228

**Published:** 2021-09-10

**Authors:** Mojtaba Madadi Asl, Saeideh Ramezani Akbarabadi

**Affiliations:** 1 Department of Physics, Institute for Advanced Studies in Basic Sciences (IASBS), Zanjan, Iran; 2 Department of Physics, University of Guilan, Rasht, Iran; Pandit Deendayal Petroleum University, INDIA

## Abstract

Synaptic strengths between neurons in brain networks are highly adaptive due to synaptic plasticity. Spike-timing-dependent plasticity (STDP) is a form of synaptic plasticity induced by temporal correlations between the firing activity of neurons. The development of experimental techniques in recent years enabled the realization of brain-inspired neuromorphic devices. Particularly, magnetic tunnel junctions (MTJs) provide a suitable means for the implementation of learning processes in molecular junctions. Here, we first considered a two-neuron motif subjected to STDP. By employing theoretical analysis and computer simulations we showed that the dynamics and emergent structure of the motif can be predicted by introducing an effective two-neuron synaptic conductance. Then, we considered a phenyl-based single-molecule MTJ connected to two ferromagnetic (FM) cobalt electrodes and investigated its electrical properties using the non-equilibrium Green’s function (NEGF) formalism. Similar to the two-neuron motif, we introduced an effective spin-polarized conductance in the MTJ. Depending on the polarity, frequency and strength of the bias voltage applied to the MTJ, the system can learn input signals by adaptive changes of the effective conductance. Interestingly, this voltage-dependent plasticity is an intrinsic property of the MTJ where its behavior is reminiscent of the classical temporally asymmetric STDP. Furthermore, the shape of voltage-dependent plasticity in the MTJ is determined by the molecule-electrode coupling strength or the length of the molecule. Our results may be relevant for the development of single-molecule devices that capture the adaptive properties of synapses in the brain.

## Introduction

Synaptic plasticity is a fundamental brain mechanism for learning and information storage [1, 2]. By activity-dependent modification of the synaptic strengths, synaptic plasticity plays a key role in shaping neural circuits during development [3] and its dysfunction may be involved in several neuropsychiatric disorders [4]. The modern notion of long-term synaptic changes was emerged when Hebb [5] proposed that the synaptic connection between a pair of pre- and postsynaptic neurons is strengthened by their correlated firing activity, later termed as the Hebbian learning [6]. Hebb’s postulate was then extended to incorporate the weakening of synapses between neurons with uncorrelated firing activity [7]. In this context, the persistent strengthening of the synaptic efficacy is called long-term potentiation (LTP), whereas its weakening is termed as long-term depression (LTD). Inspired by the Hebb’s idea, numerous experiments were devoted to validate long-term activity-dependent synaptic changes.

In particular, stimulus pairing experiments studying synaptic plasticity observed that long-term synaptic changes may causally depend on the spike times of pre- and postsynaptic neurons [8, 9]. This form of synaptic plasticity is called spike-timing-dependent plasticity (STDP) [8–10]. The classical STDP is characterized by a temporally asymmetric learning window [9, 11], such that when the presynaptic spike precedes the postsynaptic spike (i.e. causal firing or positive timing), the corresponding synapse undergoes LTP, whereas LTD of the synapse is induced in the reverse scenario (i.e. non-causal firing or negative timing) [8]. In this way, the interplay between neuronal activity and synaptic connectivity ultimately determines the emerging structure and dynamics between neurons [12]. However, the classical STDP suffers some drawbacks, e.g., it fails to account for the emergence of strong bidirectional connections in cortex [13, 14]. Later, it was suggested that a model of voltage-dependent STDP where the change of the synaptic strengths depends on the postsynaptic membrane potential can explain experimentally observed connectivity patterns in cortex [15, 16].

Experimental advances provided a means for the realization of neuromorphic structures designed to mimic the behavior of synapses. Brain-inspired learning processes are widely demonstrated, e.g., in memristor-based systems as adaptive memory elements [17, 18]. In fact, memristive devices can serve as brain-like synapses implemented in neuromorphic systems characterized by resistance (or equivalently conductance) switching behaviors [19–21]. Experimentally, memristive devices showing synaptic plasticity properties can be realized in different platforms. For instance, short-term plasticity, long-term plasticity or STDP were demonstrated in the synaptic behavior of metal/conducting polymer/metal structures [22], metal/insulator/metal magnetic tunnel junctions (MTJs) [20], metal-metal double layers [21], oxide-based devices [23] and organic polymers [24] as memristive systems. Motivated by these observations, theoretical/computational studies used simple models to capture adaptive synaptic behavior of memristive devices for neural network applications [25, 26].

Low-dimensional molecular junctions offer unique functional properties in comparison to bulk materials. One of the most appealing example of a functional molecule is the molecular switch [27]. In this context, single-molecule switches realized by, e.g., scanning tunneling microscope break junction (STM-BJ) [27, 28] or mechanically controllable break junction (MCBJ) [29, 30] techniques are promising candidates for future neuromorphic devices [31]. Single-molecule switches are governed by various mechanisms including light emission [32], electric field application [33], or temperature variations [34]. These variables can trigger the corresponding switching mechanism between different multistable states, e.g., molecular conformations, dipole orientations or charge states [27]. In MTJs, for example, conductance switching can occur due to the switching of spin polarization between spin-up and spin-down states triggered by the bias voltage applied to the MTJ [35, 36].

MTJs provide a well-characterized mechanism for the implementation of learning process in spintronic devices [37, 38], due to their rapid and high-density information storage capabilities as nonvolatile magnetic random access memory (MRAM) [39]. Theoretical and experimental studies employed fundamental effects observed in MTJs, e.g. tunneling magnetoresistance (TMR) arising from the difference of the electronic density of states (DOS) at the Fermi level between spin-up and spin-down states, to examine spin-polarized transport properties of single-molecule MTJs and its application in brain-inspired neuromorphic computing [35, 40]. It has been suggested that MTJs can artificially mimic neurophysiological properties of synapses [20] and neurons [41], possibly through voltage-controlled spintronic mechanisms and conductance switching behavior of the system [42].

MTJs can show bistability in the spin-polarized conductance due to the switching of spin polarization [35, 36]. Hence, we hypothesized that a two-terminal MTJ may be able to show some kind of plasticity in its spin-polarized conductance profile, similar to those observed in neuronal systems. To test this idea, we took a multidisciplinary approach and divided the study into two parts. In the first part, we considered a two-neuron motif and investigated its dynamics and emergent structure by theoretical analysis and computer simulations when the synaptic strengths were modified according to the classical STDP rule. Without loss of generality and for analytical tractability, we used a simple model of type-II neuronal phase oscillators that can capture the dynamics of spiking neuron models such as the Hodgkin-Huxley model [43]. The motivation behind this part was to illustrate that introducing a plasticity-induced effective synaptic strength, i.e., the ratio of relative synaptic strengths to their sum in the two-neuron motif can explain the emergent structure and dynamics between a pair of neurons subjected to STDP [43, 44]. Our results indicated that strong symmetric connections (i.e. bidirectional loops) can form nearly in-phase or anti-phase synchronization between neurons, whereas asymmetric (i.e. unidirectional) connections lead to a mixture of phase lag distribution.

Then, in the second part we considered a single-molecule MTJ comprising a phenyl dithiol (PDT) molecule or a biphenyl dithiol (BPDT) molecule connected to two ferromagnetic (FM) cobalt electrodes mediated by a sulfur anchoring unit and inspected current-voltage characteristics and conductance using the non-equilibrium Green’s function (NEGF) formalism. PDT and BPDT molecules are aromatic organic compounds characterized with *π* orbitals suggesting that they are suitable candidates for transport studies [45]. To test our idea regarding the qualitative similarity between the neuron system and MTJ, we introduced an effective spin-polarized tunneling conductance, i.e. the ratio of relative conductances in parallel and anti-parallel configurations to their sum, similar to that defined in the two-neuron motif. Interestingly, we noticed that the change of this two-component spin-polarized conductance crucially depends on the bias voltage applied to the MTJ where its behavior is reminiscent of the classical STDP characterized with a temporally asymmetric learning window. Accordingly, we named this behavior as the voltage-dependent plasticity of the spin-polarized conductance.

To illustrate that the effective spin-polarized conductance is capable of learning (similar to the neuronal system), we applied sinusoidal voltage signals to the MTJ and calculated the subsequent changes in the effective spin-polarized conductance. The results indicated that the MTJ can learn or forget the input signal via changing its conductance in an LTP-/LTD-like manner, depending on the polarity, frequency and strength of the applied voltage. Furthermore, we showed that the shape of the voltage-dependent plasticity of conductance is determined by the modulatory effect of the coupling strength between the molecule and the electrodes or the length of the phenyl-based molecule. Our results may be relevant for the development of single-molecule neuromorphic devices engineered based on the adaptive properties of synapses in brain networks.

## Materials and methods

### Neuronal phase oscillator model

**Fig 1:**
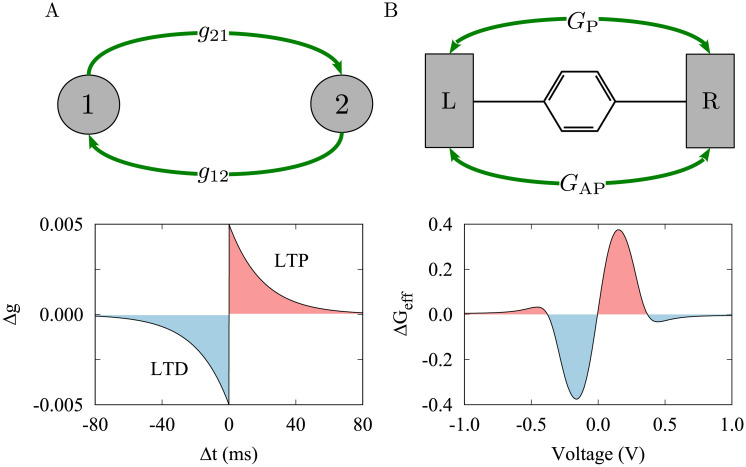
Schematic representation of the two-neuron motif and MTJ. (**A**) (Top) Two neuronal phase oscillators (1/2) reciprocally connected via synapses of strength *g*_21_/*g*_12_. (Bottom) The synaptic change governed by the classical temporally asymmetric STDP profile. STDP parameters were *A*_+_ = *A*_−_ = 0.005 and *τ*_+_ = *τ*_-_ = 20 ms. (**B**) (Top) A single-molecule MTJ composed of a PDT molecule connected to two FM cobalt electrodes (L/R) characterized by tunneling conductance in parallel (*G*_P_) and anti-parallel (*G*_AP_) configurations. (Bottom) The derivative of the effective conductance is calculated to provide a measure of its change.

Two reciprocally connected excitatory neuronal phase oscillators were considered to construct the two-neuron motif schematically shown in Fig 1A (top). The general form of the reduced phase model for weakly pulse-coupled neuronal oscillators is given by [43]:

$$\begin{array}{c} \dot{\varphi_{i}}=\omega_{i}+\frac{1}{2\pi }\sum_{j=1}^{N}g_{ij}\;Z\left(\psi +\varphi_{i}-\varphi_{j}\right),\;\;i,j=1,2,...,N;\;\left(j\neq i\right), \end{array}$$ (1)

where *ω*_*i*_ is the frequency of the oscillator and *Z*(*φ*) is the infinitesimal phase sensitivity. The neuronal oscillators are connected to each other via synapses of strength *g*_*ij*_ with transmission delay (*ψ*). *φ*_*i*_ represents the phase of the *i*-th oscillator and *N* is the number of oscillators. The neurons fire once their phase crosses multiples of 2*π*. The evolution of the phase lag (*χ* = *φ*_2_ − *φ*_1_) between two type-II oscillators characterized by analytical phase response curve *Z*(*φ*) = −sin(*φ*) can be calculated by writing Eq (1) for a two-neuron motif:

$$\begin{array}{c} \begin{array}{c} \dot{\varphi_{1}}=\omega_{1}-\frac{g_{12}}{2\pi }\sin\left(\psi +\varphi_{1}-\varphi_{2}\right),\\ \dot{\varphi_{2}}=\omega_{2}-\frac{g_{21}}{2\pi }\sin\left(\psi +\varphi_{2}-\varphi_{1}\right), \end{array} \end{array}$$ (2)

and then, subtracting these coupled differential equations to obtain:

$$\begin{array}{c} \dot{\chi }=\Omega +\frac{1}{2\pi }[g_{12}\sin\left(\psi -\chi \right)-g_{21}\sin\left(\psi +\chi \right)], \end{array}$$ (3)

where Ω = *ω*_2_ − *ω*_1_ is the frequency mismatch of neurons. Without loss of generality, one can assume that the frequency mismatch between the two oscillators is negligible, i.e. Ω ≈ 0. Then, by setting $\dot{\chi }=0$ and using the trigonometric identity sin(*x* ± *y*) = sin *x* cos *y* ± cos *x* sin *y*, the phase lag fixed point can be calculated as follows [43]:

$$\begin{array}{c} \chi^{*}=\tan^{-1}(-\frac{(g_{21}-g_{12})\tan\psi }{g_{21}+g_{12}}), \end{array}$$ (4)

where *g*_*ij*_ with *i*, *j* = 1, 2(*j* ≠ *i*) is the strength of synapse connecting the presynaptic neuron *j* to the postsynaptic neuron *i*.

### Spike-timing-dependent plasticity

The synaptic strengths between neurons were modified according to the following classical temporally asymmetric STDP rule shown in Fig 1A (bottom) [9]:

$$\begin{array}{c} \Delta g=A_{\pm }\text{sgn}\left(\Delta t\right)\exp(-|\Delta t|/\tau_{\pm }), \end{array}$$ (5)

where *A*_+_(*A*_−_) and *τ*_+_(*τ*_−_) are the rate and the effective time window of synaptic potentiation (depression), respectively. The synaptic strengths were confined in the interval [*g*_min_, *g*_max_] = [0, 1]. The synaptic strengths were set to *g*_min_ (*g*_max_) once they cross the lower (upper) limit of their allowed range.

### Magnetic tunnel junction model

The molecular junction is composed of a phenyl-based molecule connected to two FM cobalt electrodes schematically shown in Fig 1B (top). The molecular junction is described by the wide-band (WB) approximation in the non-equilibrium Green’s function (NEGF) formalism. The retarded Green’s function of the molecule connected to the FM electrodes in terms of spin state (*σ* = ↑/↓) can be expressed as follows:

$$\begin{array}{c} G^{r,\sigma }\left(\varepsilon \right)={\left(\varepsilon I-H_{M}-\Sigma_{L}^{\sigma }-\Sigma_{R}^{\sigma }\right)}^{-1}, \end{array}$$ (6)

where *H*_M_ is the Hamiltonian of the molecule that is described by the Hückel formalism:

$$\begin{array}{c} H_{M}=\frac{C}{2}S_{ij}\left(H_{ii}+H_{jj}\right), \end{array}$$ (7)

where *C* = 1.75 is the Hückel coefficient, *S*_*ij*_ indicates the overlap integral of the molecular orbitals, and *H*_*ii*_ and *H*_*jj*_ are the ionization energies of the orbitals. The transmission coefficient were calculated by the Landauer-Büttiker transmission formalism expressed in terms of the Green’s function:

$$\begin{array}{c} T^{\sigma }\left(\varepsilon \right)=\text{Tr}\left[G^{r,\sigma }\left(\varepsilon \right)\Gamma_{L}^{\sigma }\left(\varepsilon \right)G^{a,\sigma }\left(\varepsilon \right)\Gamma_{R}^{\sigma }\left(\varepsilon \right)\right], \end{array}$$ (8)

where **G**^r^(*ε*) is the retarded Green’s function given by Eq (7). The advanced Green’s function can be obtained by the relation **G**^a,*σ*^(*ε*) = [**G**^r,*σ*^(*ε*)]^†^. $\Gamma_{\alpha }^{\sigma }=-2\;\text{Im}\Sigma_{\alpha }^{\sigma }$ is given in terms of the self-energy $\Sigma_{\alpha }^{\sigma }$ of the lead *α* which mediated the coupling between the molecule and the electrodes as follows:

$$\begin{array}{c} \Sigma_{L/R}^{\sigma }=-\frac{i}{2}\left(2\pi \right|\gamma_{L/R}{|}^{2}\rho_{L/R}^{\sigma }), \end{array}$$ (9)

where $i^{2}=\sqrt{-1}$, *γ*_L/*R*_ is the coupling strength between the molecule and the electrodes (here we assumed *γ*_L_ = *γ*_R_ = *γ*), and $\rho_{L/R}^{\sigma }$ is the DOS of the left (L) or right (R) electrode in terms of the spin *σ* which is independent of the applied voltage and energy in the WB approximation used in this study. The DOS for the spin-up and spin-down states of the cobalt electrodes were chosen as *ρ*↑ = 0.5772 1/eV and *ρ*↓ = 0.1367 1/eV, respectively [45].

In the Landauer-Büttiker formalism, the current is given by:

$$\begin{array}{c} I^{\sigma }=\frac{e}{h}\int T^{\sigma }\left(\varepsilon \right)[f_{L}\left(\varepsilon \right)-f_{R}\left(\varepsilon \right)]d\varepsilon , \end{array}$$ (10)

where *e* = 1.6 × 10^−19^ C is the charge of electron, and *h* = 6.626 × 10^−34^ m^2^kg/s is the Planck constant. *f*_L/R_(*ε*) is the Fermi distribution function of the left (L) or right (R) electrode with chemical potential, *μ*_L/R_ = *ε*_F_ ± *eV*/2, where *ε*_F_ is the Fermi energy. The Fermi distribution function is given by:

$$\begin{array}{c} f_{L/R}\left(\varepsilon \right)=\frac{1}{\exp(\left(\varepsilon -\mu_{L/R}\right)/k_{B}T)+1}, \end{array}$$ (11)

where *k*_B_ = 1.38 × 10^−23^ JK^−1^ is the Boltzmann constant.

## Results

### Two-neuron motif

We first considered a two-neuron motif schematically shown in Fig 1A (top) comprising two neuronal phase oscillators described by Eq (1) connected to each other via plastic synapses where their strength is modified by the classical STDP introduced in Eq (5). The fixed point of phase lag between neurons (χ*) is given by Eq (4) that can be rearranged to χ* = tan^−1^(−*g*_eff_ tan*ψ*) by introducing an effective synaptic conductance (*g*_eff_), i.e. the ratio of relative synaptic strengths to their sum, as follows [44]:

$$\begin{array}{c} g_{\text{eff}}=\frac{g_{12}-g_{21}}{g_{12}+g_{21}}, \end{array}$$ (12)

**Fig 2:**
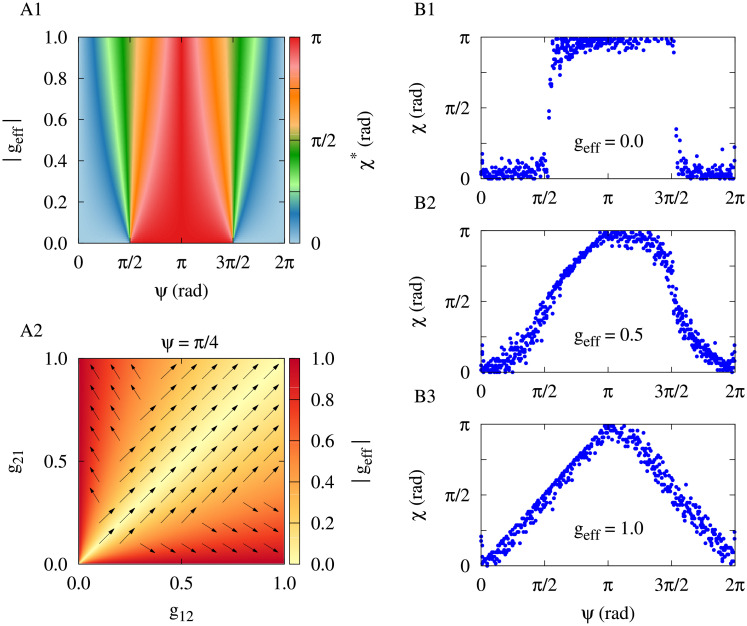
Theoretical and simulation results for the two-neuron motif. (**A1**) Theoretical prediction of the fixed point of phase lag between the two neurons in terms of the effective synaptic strength and delay calculated by Eq (4). (**A2**) Theoretical prediction of the two-neuron synaptic change (arrows) in terms of the effective synaptic strength at a given delay (indicated above panel) calculated by Eq (5). (**B1-B3**) Simulation results of the steady-state phase lag between neurons for different values of the effective synaptic strength (denoted in the figure) are consistent with the theoretical predictions.

The evolution of the phase lag between spike times of neurons (i.e. the dynamics of the motif) can be theoretically predicted by calculating the phase lag fixed point given by Eq (4) in terms of the *g*_eff_ and transmission delay (*ψ*) as shown in Fig 2A1. Theoretical analysis suggests that depending on the value of *g*_eff_ and the range of delay the phase lag between neurons can attain two extreme regimes, i.e. in-phase state (zero phase lag) or anti-phase state (*π* phase lag).

The theoretically predicted direction of change of the synaptic strengths calculated from Eq (5) is depicted in Fig 2A2 (arrows) for an examplary value of delay (i.e. *ψ* = *π*/4) showing that in this case the two-neuron motif is more likely to attain a strong bidirectional loop, i.e. *g*_eff_ = 0.0. Simulation results presented in Fig 2B1–2B3 are consistent with the theoretical predictions. When the change of the synaptic strengths occurs in a symmetric manner, i.e. *g*_eff_ = 0.0 (Fig 2B1), the steady-state phase lag between neurons asymptotically approaches to its theoretically predicted fixed point in Fig 1A1 to establish either an in-phase state or an anti-phase state depending on the range of delay. However, when the synaptic strengths are modified asymmetrically, i.e. *g*_eff_ = 1.0 (Fig 2B3), the phase lag linearly depends on the transmission delay comprising middle-range phase lags. When the synaptic strengths are modified according to STDP, the fixed point of phase lag and the effective synaptic conductance compete to determine the ultimately evolving dynamics and structure of the motif [43].

#### Activity-dependent learning in the two-neuron motif

**Fig 3:**
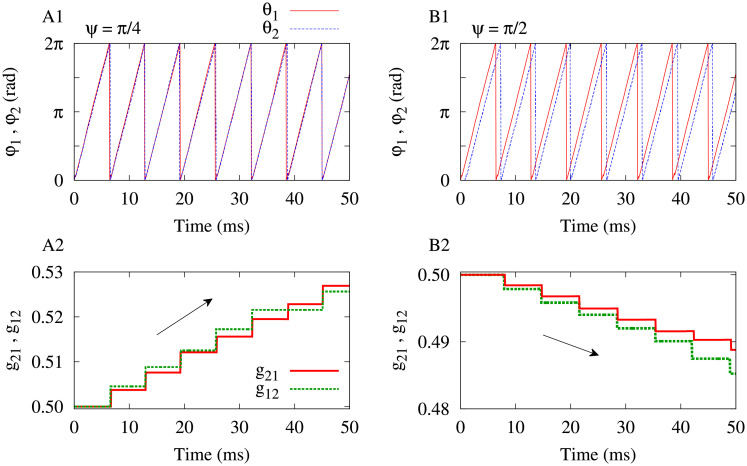
Activity-dependent learning in the two-neuron motif by STDP. (**A1,A2**) Activity of the neurons in terms of the phase of oscillations characterized with strong phase synchronization (*χ* ≈ 0), when delay is *ψ* = *π*/4 (A1), and the corresponding LTP of the synapses (A2). (**B1,B2**) Activity of the neurons in terms of the phase of oscillations associated with a loosely synchronized state (i.e. small non-zero *χ*), when delay is *ψ* = *π*/2 (B1), and the corresponding LTD of the synapses (B2).

In the two-neuron motif, STDP adjusts the synapses between the two neurons based on their activity. Therefore, as shown in Fig 3 the two-neuron motif undergoes learning, i.e, LTP/LTD of synapses due to the correlated/uncorrelated activity of neurons. The extent of learning depends on various parameters, depending on the system. Here, as argued in the discussion of Fig 2, the transmission delay between the neurons crucially determines the dynamics (phase lag between neurons) and structure (strength of synapses) of the motif.

When *ψ* = *π*/4, the firing activity of neurons is characterized with strong phase synchronization, i.e., *χ* ≈ 0 in Fig 3A1 (also see Fig 2). In this range of parameters, the STDP rule leads to the LTP of both synapses between neurons and shapes a strong bidirectional loop. This is illustrated in Fig 3A2 where both the synaptic strengths are strengthened. On the contrary, as shown in Fig 3B1 when the transmission delay is increased to *ψ* = *π*/2 the activity of the neurons is shifted to a loosely phase synchronized state (i.e., small non-zero *χ*). This ultimately leads to the LTD of synapses where both the synaptic strengths are weakened (see Fig 3B2).

### Magnetic tunnel junction

**Fig 4:**
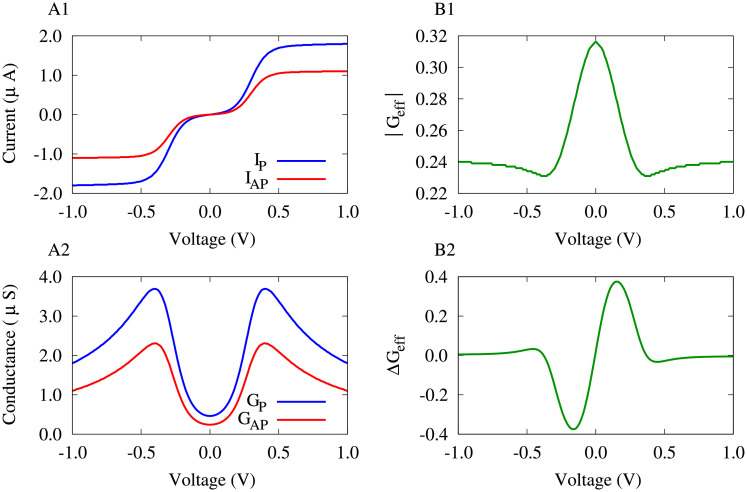
Voltage-dependent plasticity of effective conductance in the PDT-based MTJ. (**A1**) Current-voltage characteristics for parallel and anti-parallel configurations. (**A2**) The corresponding tunneling conductance derived from the spin-polarized current for parallel and anti-parallel states. (**B1**) The effective tunneling conductance introduced by Eq (13). (**B2**) The derivative of the effective conductance is calculated to provide a measure of its change which is reminiscent of the classical asymmetric STDP profile. The molecule-electrode coupling strength was *γ* = 0.1.

From a neurophysiological perspective, the synaptic strengths are representative of the conductance strength of the ion channels participating in the transmission of neurotransmitters at synaptic connections between neurons. Comparatively, we considered a single-molecule MTJ consisted of a PDT molecule connected to two FM cobalt electrodes mediated by a sulfur anchoring unit schematically shown in Fig 1B (top). The current-voltage characteristics of the MTJ is shown in Fig 4A1 for the spin parallel (*I*_P_; when the tunneling occurs by majority/minority carriers from both electrodes) and anti-parallel (*I*_AP_; the tunneling occurs by majority carriers from one electrode and minority carriers from the other one) configurations. This leads to two independent conduction channels for spin-up and spin-down states.

Both parallel and anti-parallel currents are close to zero near zero bias voltages, however, when a bias voltage is applied to the junction, the chemical potential of the electrodes is changed such that frontier molecular energy levels, either the highest occupied molecular orbital (HOMO) or the lowest unoccupied molecular orbital (LUMO), are adjusted within the bias window. Hence, the current is increased with bias voltage in a step-like manner such that the current is relatively constant for a range of bias voltages and is then increased when the potential energy barrier is overcame. This is due to the occupation of molecular levels and the appearance of new channels for electron transfer [35]. Another notable observation is that the parallel current is greater than the anti-parallel current which can be traced back to the definition of the parallel and anti-parallel configurations ascribed to the low tunneling resistance (parallel spin) and high tunneling resistance (anti-parallel spin) states [46].

The parallel and anti-parallel conductances of the MTJ are depicted in Fig 4A2 which were calculated from the corresponding spin-polarized currents that expectedly show significant peaks around those bias voltages where the current was increased. In correspondence to the effective synaptic strength defined for the two-neuron motif in Eq (12), we introduced an effective spin-polarized tunneling conductance, i.e. the ratio of relative conductances in spin parallel *G*_P_) and anti-parallel (*G*_AP_) configurations to their sum, as follows:

$$\begin{array}{c} G_{\text{eff}}=\frac{G_{\text{AP}}-G_{P}}{G_{\text{AP}}+G_{P}}, \end{array}$$ (13)

The idea of introducing a two-component conductance was captured from the neuronal system and can establish a relation between the two-neuron motif (*g*_eff_ in Eq (12) as a function of *g*_21_ and *g*_12_) and the MTJ (*g*_eff_ in Eq (13) as a function of *G*_P_ and *G*_AP_). In addition, as previously argued in the discussion of Fig 3, the LTP/LTD of the synapses in the two-neuron motif occurs due to the polarity (positive/negative timing) of phase lag and the range of transmission delay between neurons. The phase oscillations of neurons considered here can be mapped into the oscillations of membrane voltage of the phenomenological neuron models. By the same token, as we will show below the LTP-/LTD-like strengthening/weakening of effective conductance in the MTJ occurs according to the polarity of the applied bias voltage (see Fig 7).

The quantity *G*_eff_ is plotted in Fig 4B1 which shows a peak around the zero bias voltage. It is more interesting to take a look at the derivative of the effective tunneling conductance (Δ*G*_eff_) depicted in Fig 4B2 that shows an asymmetric change of *G*_eff_ with respect to the bias voltage. The peaks of the change of *G*_eff_ correspond to the current increasing jump in Fig 4A1 and 4A2. Interestingly, the change of the *G*_eff_ is similar to the classical STDP profile characterized with a temporally asymmetric learning window (see Fig 1A, bottom), but here, Δ*G*_eff_ depends on the bias voltage applied to the MTJ. The change of Δ*G*_eff_ for positive/negative bias voltages in Fig 4B2 resembles LTP/LTD of the synaptic strengths in Fig 1A (bottom), respectively.

#### Molecule-electrode coupling strength shapes plasticity in the MTJ

**Fig 5:**
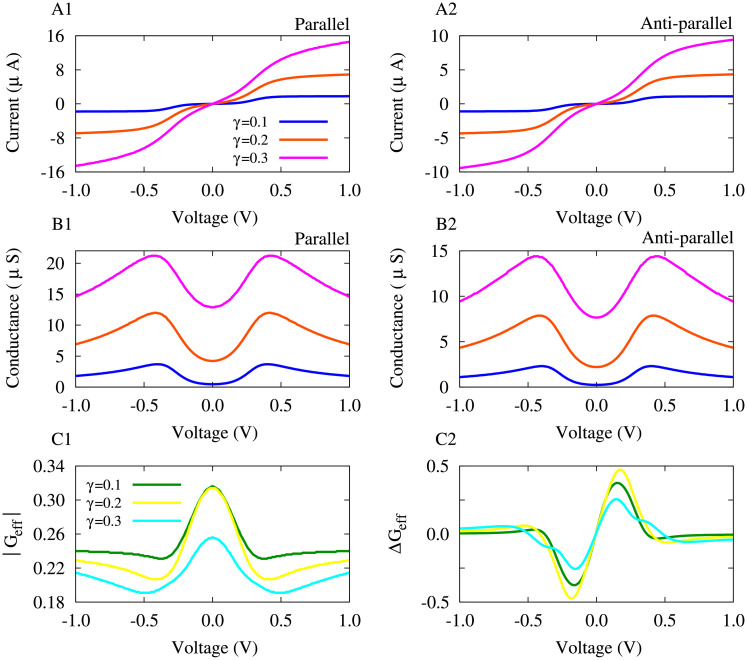
The effect of molecule-electrode coupling strength on the voltage-dependent plasticity of effective conductance in the PDT-based MTJ. (**A1,A2**) Current-voltage characteristics in parallel (A1) and anti-parallel (A2) configurations for different values of the molecule-electrode coupling strength. (**B1,B2**) The corresponding tunneling conductance derived from the spin-polarized current for parallel (B1) and anti-parallel (B2) states. (**C1**) The effective tunneling conductance is calculated when the molecule-electrode coupling strength is varied. (**C2**) The derivative of the effective conductance as a measure of its asymmetric change.

The current-voltage characteristics of the MTJ can be shaped by modification of coupling between the molecule and the electrodes which is mediated by the molecule-electrode coupling strength (*γ*) introduced in Eq (9). To address how the molecule-electrode coupling strength can affect the shape of the voltage-dependent plasticity of *G*_eff_ in the PDT-based MTJ, we varied *γ* and depicted the spin-polarized current, effective conductance and its change in Fig 5.

Both the parallel (Fig 5A1) and anti-parallel (Fig 5A2) spin-polarized currents are increased (orange/magenta curves) by increasing the molecule-electrode coupling strength compared with the case where *γ* = 0.1 (blue curve). Expectedly, the corresponding tunneling conductance in parallel (Fig 5B1) and anti-parallel (Fig 5B2) configurations are also increased with molecule-electrode coupling strength. The molecule-electrode coupling modified *G*_eff_ such that its peak around zero bias voltage is ultimately reduced by the transition from *γ* = 0.1 (Fig 5C1, green) to *γ* = 0.3 (Fig 5C1, cyan). Finally, the overall asymmetric shape of the voltage-dependent plasticity of effective conductance is relatively robust to the changes of the molecule-electrode coupling strength as shown in Fig 5C2. However, the amplitude of the conductance change is fairly increased when *γ* = 0.2 (Fig 5C2, yellow) and then decreased when *γ* = 0.3 (Fig 5C1, cyan). These modulatory effect of the molecule-electrode coupling strength on the shape of voltage-dependent plasticity of effective conductance an be attributed to the changes of the current-voltage characteristics. In fact, *γ* modifies the window required for current flow through the MTJ by the realignment of the energy separation between HOMO and LUMO levels [47], i.e. the HOMO − LUMO gap, and therefore, the resultant conductance change is reshaped.

#### Length-dependent modulation of plasticity in the MTJ

**Fig 6:**
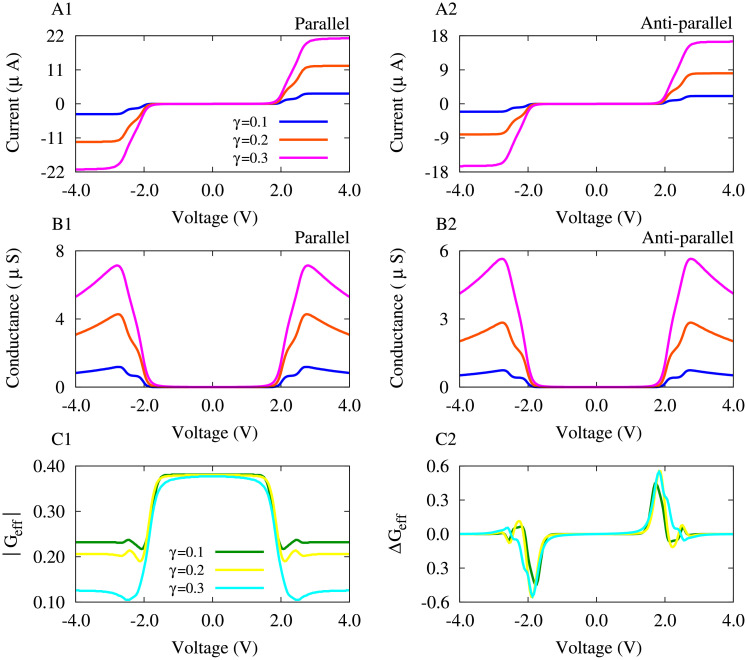
The length of the molecule determines the voltage-dependent plasticity of effective conductance. (**A1,A2**) Current-voltage characteristics in parallel (A1) and anti-parallel (A2) configurations for different values of the molecule-electrode coupling strength in the BPDT-based MTJ. (**B1,B2**) The corresponding tunneling conductance derived from the spin-polarized current for parallel (B1) and anti-parallel (B2) states. (**C1**) The effective tunneling conductance is calculated when the molecule-electrode coupling strength is varied. (**C2**) The derivative of the effective conductance as a measure of its asymmetric change.

In order to inspect how the length of the central molecule in the MTJ can modulate the shape of the voltage-dependent plasticity of effective conductance, we repeated our calculations for the BPDT molecule (characterized with two phenyl rings) in Fig 6 and compared the results with those obtained from the PDT molecule (characterized with a single phenyl ring) presented in Fig 5. The HOMO − LUMO gap in the BPDT molecule is increase compared to the PDT molecule, and hence, both the parallel (Fig 6A1) and anti-parallel (Fig 6A2) spin-polarized currents are zero for greater values of the bias voltage around zero and the step-like increase of currents is pushed towards greater bias voltages. This effect is also appeared in parallel (Fig 6B1) and anti-parallel (Fig 6B2) conductances.

The effective conductance show a wide peak around the zero bias voltage where it is slightly decreased by increasing the molecule-electrode coupling strength (see Fig 6C1). The main result of the increasing the length of the molecule in the BPDT-based MTJ was to extend the voltage-dependent strengthening/weakening of conductance towards greater bias voltages shown in Fig 6C2. Around the zero bias voltage the *G*_eff_ remains unchanged and it is suddenly increased/decreased near *V* = 2.0 V. However, in the PDT-based molecule the change of the *G*_eff_ is most significant around *V* = 2.0 V (see Fig 5C2).

In addition, the effect of the molecule-electrode coupling strength on current-voltage characteristics, conductance, and the voltage-dependent plasticity of the BPDT-based MTJ is qualitatively similar to the effects observed in the PDT-based MTJ. Increasing the molecule-electrode coupling strength from *γ* = 0.1 (Fig 6A1–6B2, blue) to *γ* = 0.3 (Fig 6A1–6B2, orange/magenta) increased the spin-polarized current and conductance. The *G*_eff_ is modulated by increasing the molecule-electrode coupling strength (Fig 6C1) such that the amplitude and duration of the strengthening/weakening of conductance in Fig 6C2 is relatively increased for *γ* = 0.2 and *γ* = 0.3 in comparison to the case where *γ* = 0.1.

#### Voltage-dependent learning in the MTJ

In the two-neuron motif, the LTP/LTD of synapses due to STDP depends on the temporal correlation of the activity of neurons (or alternatively, the phase lag between their oscillations). In the MTJ, however, the LTP-/LTD-like behavior is an intrinsic property of the system (due to the switching of spin polarization between spin-up and spin-down states) and depends on the polarity of the applied bias voltage.

**Fig 7:**
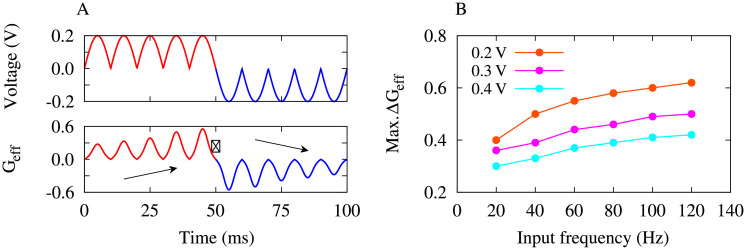
Voltage-dependent learning in the MTJ. (**A**) (Top) A sinusoidal voltage signal with amplitude (strength) *V*_max_ = 0.2 V and frequency *ω* = 100 Hz is applied to the MTJ. (Bottom) Calculated effective conductance of the MTJ in response to the applied voltage. Arrows show LTP-like and LTD-like behavior of the effective conductance depending on the polarity of the input signal. (B) Frequency dependency of learning in the MTJ for different values of the signal strength. The molecule-electrode coupling strength was *γ* = 0.1.

To further demonstrate that the voltage-dependent plasticity of effective conductance in the MTJ undergoes a form of learning process, a sinusoidal voltage signal with amplitude (strength) *V*_max_ = 2.0 V and frequency *ω* = 100 Hz was applied to the MTJ and the subsequent changes in the effective spin-polarized conductance were calculated. The results presented in Fig 7 show that the MTJ can learn or forget the applied signal based on the polarity of the voltage. In this way, positive applied voltage (Fig 7, top panel, red curve) leads to a LTP-like strengthening of the effective conductance (Fig 7, bottom panel, red curve). On the contrary, negative applied voltage (Fig 7, top panel, blue curve) results in a LTD-like weakening of the effective conductance (Fig 7, bottom panel, blue curve).

Furthermore, to inspect the dependency of the voltage-dependent plasticity of effective conductance on the frequency or amplitude (strength) of the input signal we used three different input signals characterized by voltage strengths *V*_max_ = 0.2, 0.3, 0.4 V and calculated the resultant (maximum) change in the *G*_eff_ for different input frequencies (Fig 7B). The results indicate that Δ*G*_eff_ is generally increased (and relatively saturated) with increasing the frequency of the input signal, regardless of the signal strength (Fig 7B). However, according to the voltage-dependent plasticity profile in Fig 4B2, the maximum of Δ*G*_eff_ occurs at ∼ ±0.2 V. This is verified in Fig 7B (orange curve), where increasing the strength of the bias voltage relatively suppressed the maximum of Δ*G*_eff_ in Fig 7B (magenta/cyan curve).

## Discussion

In summary, here by establishing a correspondence between the synaptic strengths in a two-neuron motif, and parallel and anti-parallel conductances in a two-terminal MTJ we showed that voltage-dependent changes of the introduced effective tunneling conductance in the MTJ resembles the plasticity-induced changes of the synaptic strengths between the two neurons. In the two-neuron motif, *G*_eff_ can account for the emergent dynamics and structure between neurons: A perfectly symmetric connection (i.e. *g*_eff_ = 0.0) leads to in-phase or anti-phase synchronization states, whereas a perfectly asymmetric connection (i.e. *g*_eff_ = 1.0) results in a mixed states of in-phase and anti-phase lags. The emergence of these states is a direct consequence of STDP that leads to activity-dependent LTP/LTD of the synapses in the two-neuron motif.

In the MTJ, however, the change of the effective conductance undergoes a voltage-dependent plasticity profile that is reminiscent of the classical temporally asymmetric STDP profile. This voltage-dependent plasticity is an intrinsic property of the MTJ and enables the system to learn or forget particular input signals by LTP-/LTD-like strengthening/weakening of the conductance. The extent of learning in the MTJ depends on the frequency and strength of the input signal. Furthermore, modification of the MTJ, e.g. changing the strength of the molecule-electrode coupling or the length of the central molecule connected to the FM electrodes, can modulate the shape of the voltage-dependent plasticity.

In this study we used the thiol anchoring unit to mediate the coupling of the PDT and BPDT molecules to the FM cobalt electrodes. However, it was previously shown that the electron donating/accepting character of anchoring units can modify the conductance of single-molecule junctions by the energy-level line up relative to the Fermi energy of electrodes [48–50], ultimately resulting in an enhanced/suppressed conductance depending on the nature of the molecular structure [51, 52]. Furthermore, here we used unperturbed PDT and BPDT molecules as the central molecule in the MTJ. However, functionalization of the molecule with side groups can also have dramatic modulatory effects on the conductance of the system by reorganization of the frontier molecular orbitals relative to the Fermi energy level of the electrodes [52, 53]. These modifications can favorably or unfavorably tune the behavior of the voltage-dependent plasticity of effective conductance in MTJ which certainly deserves further examination.

Our results indicated that the optimized voltage-dependent plasticity of the effective spin-polarized tunneling conductance can be controlled by the length of the central molecule or likely by its chemical nature. However, the asymmetry of this voltage-dependent plasticity of conductance is determined by the FM electrodes, and hence, can be controlled by chemical engineering of the junction. Employing other types of symmetric/asymmetric FM electrodes may crucially modulate the shape of voltage-dependent plasticity. Such modulations may provide an optimized framework to inspect voltage-controlled LTP/LTD in MTJs required for the artificial implementation of brain-inspired neuromorphic computing in single-molecule junctions.
